# Graduate Study on the Continent—Germany

**Published:** 1908-06-13

**Authors:** 


### HOSPITAL ADMINISTRATION.

### CONSTRUCTION AND ECONOMICS.

### GRADUATE STUDY ON THE CONTINENT—GERMANY.

V.—SPECIAL WORK IN BERLIN.

The graduate who goes abroad does so usually with the object of attacking one or more subjects as specialties. Even if he comes to gain a little more knowledge on all subjects, to see what, in its most catholic sense, Continental methods in medicine and surgery can teach him, to walk the various hospitals and to glean from every field, private as well as public; even if he has these laudable intentions, it is yet rare to find that there is no subject that attracts him beyond others.

There are very many departments in medicine and consequently very many attractions, but the student who wishes to pursue one subject more particularly than another often finds a difficulty in his ignorance of the possibilities of one or other large centre he may select. In general, it may be said that every large medical centre, Paris, London, Vienna, and Berlin offers scope and opportunities for the pursuit of study in any speciality the student may select. That statement is relatively true, but its truth is not absolute. That is to say in many cases such large centres do not offer the same scope nor the same opportunities which lie in smaller centres where one or more specialties are made much of. Thus London is hardly the centre the student would select for specialising in orthopædics; Vienna, again, does not offer much material for the study of tropical medicine; and Berlin is by no means such an excellent field for the fever specialist as London. The student will have to consider carefully what special advantages each particular centre offers, both in men and in material, for plenty of material with only an indifferent teacher is more or less a drawback.

As a centre for "specialties," Berlin is easily in the front rank. Its vast wealth of clinical material, drawn from a very wide and very populous district, is worked and controlled by men of world-wide eminence in many subjects, though here, as elsewhere, certain subjects stand out prominently while others retire into the background. Taking surgical specialties first, one ought to put at the head of the list orthopædics. During the lifetime of the late Professor Hoffa, Berlin was probably the best place for orthopædic work on the Continent. Hoffa has passed away, but his clinic remains, and his pupils bid fair to equal him in fame. At the Royal Surgical Clinic, where Professor Klapp controls the orthopædic department, at the private clinics such as that of Professor Joachimsthal, the student may see orthopædic work which for excellence and worth, as judged by results, is unsurpassed anywhere. By taking a course under one or other worker, he will obtain advantages which he certainly would not obtain elsewhere—except possibly in Vienna where the field is overcrowded with workers. Perhaps we cannot do better than outline the scheme of work in a course. Daily, every morning of the week, from 11 o'clock or earlier till 2 in the afternoon, five members in the class attend at out-patients' and diagnosis cases. The professor gives a case for diagnosis and discusses treatment which the class applies under his directions. Every day of the week, for a month, the members of the class have opportunities for putting on splints, applying plaster and celluloid bandages, and treating a variety of cases that cover practically the whole field of orthopædic work. During that time every member does a certain amount of operative work, ranging from tendon transplantation to aspirating joints and reducing dislocations. Great stress is laid on practical work, and one may characterise the course as specially adapted for the general practitioner. Conservative methods are put in the foreground. The student is shown how to treat cases which he is likely to get in private practice, and, what is especially advantageous from an educational point of view, he is shown the after results of such treatment. Equally good courses can be obtained in other departments, among which may be mentioned cystoscopy and urinary diseases; operative surgery, with special reference to the performance of operations under lumbar and local anæsthesia; ear, throat, and nose work; general surgical diagnosis and treatment, and finally, as it is a subject which is likely to attract general attention in the future, hyperæmia as a method of treatment. For bronchoscopy and special work in laryngology Berlin is probably not such a good centre as some other places, notably Freiburg and Vienna, while in general surgery, particularly intestinal work, the student will very likely compare it unfavourably with Paris and London. Still, this is individual opinion, and in any case the student will see new methods, and, what is especially worth laying stress on, he will be able to watch the results of different methods of after treatment, and thereby be in a position to select what he deems the better method for his own use later on.

In purely medical work, Berlin offers much, and it has a reputation which is fully justified. One has only to glance at the names of the various professors to recognise that special teaching is at least given by men who are well known in their respective departments for good and original work. In pediatrics Professor Heubner, Professor Baginsky, Dr. Bendix, and Dr. G. Klemperer; in pathology Professor His, Professor Salkowsky, Professor Orth, and Professor Thierfelder; in gynæcology Professor Bumm; in nervous diseases Professor Ziehen, and the still better known Dr. Oppenheim, whose private polyclinic is one of the most popular in Berlin, and Professor Remak; in pure medicine Professor Senator, Professor His, Excellenz von Leyden, Professor Kraus, and Professor Ewald; in medical jurisprudence Professor Strassmann; in bacteriology Professor Wolff; in hygiene Professor Rubner—these are only a few names out of the long list of well-known men who figure in the ranks of the professors of the University. In addition there are many private clinics, and the whole gamut of *Privat docenten*, each of the latter being a recognised specialist in the department on which he lectures. The material for study is unsurpassed, and so, too, are the facilities offered to the student. He is free to attend the clinical demonstrations, to see what goes on in the wards, and to undertake special investigations, under the supervision of the directors in the many special institutes which are nowhere so well equipped as here.

Not the least of the advantages which a study at such a large centre as Berlin brings with it is the opportunity that the student has of comparing his work with that of others in the same field, and of picking up hints from the many others who, like himself, are engaged in investigating special points. Everywhere he will meet with real courtesy and helpful encouragement. He will find that the men he comes into contact with as teachers are keen on the success of their pupils; they take a certain pride in the results obtained, results which, to a large extent, their advice and co-operation have collaborated to obtain. One caution only cannot be sufficiently impressed upon the graduate who wishes to do really good work in a special department there, and that is the advisability of acquainting himself with the German language. Only by mastering German so as to be able to read it easily, understand it as easily, and to speak it with a certain amount of fluency, can he hope to make real progress in special work. At Berlin he will find many able teachers, and the difficulties of perfecting himself in the language can easily be overcome with a little perseverance and application.

